# Effects of Topical Sesame Oil Extracted from Tahini (*Ardeh*) on Pain Severity in Trauma Patients: A Randomized Double-Blinded Placebo-Controlled Clinical Trial

**DOI:** 10.30476/BEAT.2020.82561

**Published:** 2020-07

**Authors:** Maryam Gholami, Sedigheh Torabi Davan, Maryam Gholami, Shahram Bolandparvaz, Mehrnaz Gholami, Parisa Chamanpara, Leila Shayan

**Affiliations:** 1 *Clinical Research Development Center, Nemazee Hospital, Shiraz University of Medical Sciences, Shiraz, Iran*; 2 *Transplantation Unit, Nemazee Hospital, Shiraz University of Medical Sciences, Shiraz, Iran*; 3 *Intensive Care Unit, Nemazee Hospital, Shiraz University of Medical Sciences, Shiraz, Iran*; 4 *Trauma Research Center, Department of Surgery, Shahid Rajaee (Emtiaz) Trauma Hospital, Shiraz University of Medical Sciences, Shiraz, Iran*; 5 *School of Management and Medical Information Sciences, Shiraz University of Medical Sciences, Shiraz, Iran; Cognitive Neuroscience, Institute for Cognitive Science Studies, Shahid Beheshti University, Tehran, Iran; Cognitive Science (Brain, Mind, and Education), Shahid Chamran University of Ahvaz, Ahvaz, Iran*; 6 *Trauma Research Center, Shahid Rajaee (Emtiaz) Trauma Hospital, Shiraz University of Medical Sciences, Shiraz, Iran*

**Keywords:** Sesame Oil, Traditional Persian medicine, Pain Measurement, Bruise, Emergency department

## Abstract

**Objective:**

To investigate the effects of sesame oil extracted from tahini (Ardeh) on pain severity in patients with upper or lower limbs trauma.

**Methods:**

This double-blinded randomized clinical trial study was conducted on 120 patients with upper or lower trauma in Shahid Rajaee Hospital, Shiraz, Iran, from May the 1^st^ through November 30^th^, 2016. The patients were randomly assigned to two groups using block randomization. The intervention group received topical sesame oil extracted from tahini (Ardeh) and the placebo group received cooking oil. Pain severity, pain sensitivity and heaviness of painful site were assessed.

**Results:**

Overall, we included 90 patients with traumatic limb injuries in this study who were randomized to two study groups. The mean age of the patients was 28.3 ± 6.8 (ranging from 25 to 35) years and there were 63 (70%) men and 27 (30%) women among the patients. In the sesame oil group, the mean changes in the pain severity (-1.53 ± 0.57, P<0.001), pain sensitivity (-1.45 ± 0.64, P<0.001) and heaviness of painful site (-1.56 ± 0.68, P<0.001) were significantly lower when compared to the placebo group in the second day of the intervention. None of the patients experience adverse drug effects.

**Conclusion:**

Our findings suggest that the topical use of sesame oil extracted from Tahini has a pain reliever effect on the skin after bruising and it helps prevent skin discoloration in patients with traumatic injuries of limbs.

## Introduction

A bruise is the result of smaller vessels’ bleeding into subcutaneous tissues [1, 2]. Leaking blood under the skin results in pain, swelling, and skin discoloration [3-5]. The bruise usually develops during the first 24–48 hours after the impact [1]. Pain can be defined as an unpleasant phenomenon with both sensory and emotional experience associated with actual or potential tissue damage [6]. There are some pharmacological and non-pharmacological therapies for pain. Non-pharmacological treatments can help control pain in addition to medicine [7]. Traditionally, ice has been used to reduce swelling and pain, especially because of bruising. A pack of frozen peas and an icepack or a cold compress is the forms of ice that people often apply [8]. Pain has different aspects. In the literature, pain intensity is the physical dimension of pain [9]. Also, pain sensitivity is recognized as the least experience of pain which a subject can sense [10] and pain heaviness means “the bodily sensation of weight” [11]. 

Sesame is one of the most ancient crops cultivated which has been used for more than 5 thousand years in India [12]; amongst the crop oils, it has high oil content [13, 14]. Various properties of sesame oil such as anti-inflammatory, anti-viral, anti-fungal and anti-bacterial effects make it a useful component of some pharmaceutical products [15]. The antioxidant components of sesame oil, like sesamol and sesamolin, are the main effect of this oil on the skin [16, 17]. Actually, the natural anti-oxidants have the intrinsic capabilities to prevent lipid peroxidation, which is suggested to be closely related to aging, mutation, cancer and several other diseases [18-20]. Also, this substance is useful for the prevention of oxidative damage, cardiovascular diseases, and skin tumor [21-26].

Sesame paste (also called tahini) is a complex colloidal mixture among the family of oils, which is a rich source of protein and lipid. De-hulled sesame seeds are milled and roasted without any changes in its component to make this material [27]. This material has high levels of nutrients such as calcium, iron, potassium and phosphorus, antioxidants and vitamins (B, C and E) and high content of sesame oil (more than 50%) [28]. Regarding the importance of pain management after bruising and the useful properties of sesame oil, we aimed to investigate the effect of sesame oil extracted from tahini (Ardeh) on traumatic pain of the patients referred to Shahid Rajaee Hospital in Shiraz.

## Materials and Methods

*Study population*

This double-blinded randomized placebo-controlled clinical trial study was conducted on 120 patients with upper or lower limbs trauma referred to emergency department of Shahid Rajaee Hospital, Shiraz, Iran, from May the 1st through November the 30th, 2016. All patients with one or multiple blunt trauma in the upper extremity (finger, wrist, lower arm, elbow, upper arm) or lower extremity (toe, foot, ankle, lower leg, knee, upper leg and lower trunk) occurring at least 1 hour and maximally 6 hours after admission were enrolled in the study. Inclusion criteria were age range of 15- 40 years, lack of any sign of bone fractures, internal or external bleeding, dislocation, amputation, presence of a foreign body, nerve damage, fever as well as infection, lack of cast or splint at the trauma site, lack of regional pain based on Visual Analog Scale (VAS), lack of any history of addiction, cigarette and alcohol abuse, lack of any sensitivity or allergy to the sesame plant group, not receiving drugs or herbal extracts which may interact with the study therapeutic protocol such as anti-coagulant and analgesics, and lack of diseases that may affect the pain severity such as diabetes, cardiovascular, liver, kidney, and musculoskeletal diseases. Exclusion criteria were having any sign of allergy to the sesame oil or peptic or duodenal ulcers, receiving treatment out of the study, inappropriate follow up by patients (missing follow-up more than two times), and patient’s willingness to withdraw in any phase of the study.

*Ethics and blinding*

This study was approved by the ethics committee of International Branch of Shiraz University of Medical Sciences, Iran, with the code No. IR.SUMS.REC.1394.34 and registered in the Iranian Registry of Clinical Trials (IRCT) with the code No. IRCT20171017036838N1. Before starting the interventions on patients, the researcher obtained the informed consent, verbally explained about the research, and assured the patients of confidentiality and anonymity of their data. Apart from the project coordinator, the patients, the staff involved at the clinical center, and members involved in collecting and analyzing the data were blinded to the intervention allocation. During the intervention, neither the patients nor the researcher knew which containers were treatment and placebo.

*Randomization and intervention*

Patients were randomly allocated to intervention and control groups using block randomization method. They generated 30 randomization sequences using computer-generated random numbers in permuted blocks of size four. Both groups received a 5 min light massage on the trauma site three times of a day with 8 h interval for 2 days. Patients in the intervention group received topical sesame oil extracted from tahini (Ardeh), whereas the patients in the control group just received cooking oil with the same color and container to avoid bias in the results. Also, routine cares were implemented based on the care protocol for all patients with trauma. According to this protocol, the periphery and the center of the trauma site were irrigated and cleaned with 1000 mL sterile normal saline solution (0.9%), and then dried with a sterile pad and massaged. Furthermore, based on our pilot study conducted on 10 patients in each group (not included in main sample), the topical usage of both oils showed no adverse effects. 10 drops (about 1 mm thick and 3.8 mL) of each oil extract was applied for each 50 square cm on the site of trauma, using a dropper. After that, the site of trauma was massaged lightly. 

*Study protocol*

At initial meeting considered as baseline, pain severity, pain sensitivity and the level of painful site heaviness, in addition to demographical data and the background of the NSAIDs received were assessed for both groups by the same trained specialist. Then, all the patients were taught how to take care of their trauma during 2 h in the consultation room at the recruitment hospital. All the patients were educated to put cold compress on the trauma site on the first day, put warm compress on the following days, and avoid usage of other medicines or herbal extracts for pain relief. The researcher assessed the characteristics including age and gender. For measuring the pain severity, we used Visual Analogue Scale (VAS). When responding to a VAS item, the respondents specified their level of agreement to a statement by indicating a position along a continuous line between the two end-points (0 and 10). Two other scales including pain sensitivity and heaviness of painful site were also measured. Like VAS, these two scales were measured by a continuous line between the two end-points (0 and 10) [29]. The data of this study were collected twice. Before the intervention (baseline) and after 48 hours of the intervention, pain severity, pain sensitivity and heaviness of painful site were scored in both groups. Demographical data were recorded at first. At the end of the second day of the follow up, pain severity, pain sensitivity and heaviness of painful site were assessed and scored again with the previous questionnaire. Both pre- and post-assessment and scoring were performed by the same trained specialist who was blind to the groups allocation.

*Sample Size*

To estimate the sample size, we used the result of a previous study [30] which investigated the effect of topical sesame oil on pain severity of patients with limbs trauma. This study showed a significant difference between pain severity of the patients in the sesame oil group (1.14 ± 1.43) and control group (2.83 ± 1.53) on the 10th day of the intervention. Based on these results and using the sample size formula for the two means, with a confidence level of 99% and a power of 0.99, it was estimated that 38 subjects were needed in each group. Since dropout rate might be relatively high in interventional studies and for getting more confident results, we considered 60 subjects in each group.

*Statistical Analysis*

Statistical analysis was performed using the Statistical Package for Social Sciences (SPSS Inc., Chicago, IL, USA; v.18). Descriptive statistical tests, mean, standard deviation frequency and percentage were used for demographic characteristics (Table 1). Independent sample t-test was used to compare quantitative variables between the both groups. Paired t-test was applied to compare quantitative variables before and after the intervention. Furthermore, Chi-square test was performed for qualitative variables. A *p*-value less than 0.05 was considered as statistically significance.

## Results

**Fig. 1:**
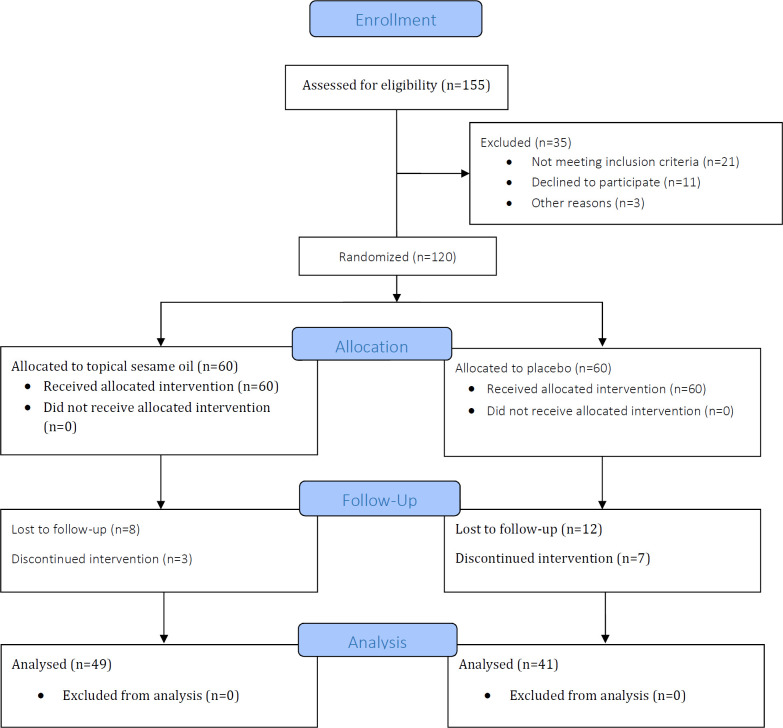
CONSORT flow diagram of the study

Overall, 120 patients with inclusion criteria participated in the study, where 10 patients were excluded during the intervention period due to NSAIDs consumption and 20 lost the follow up. Of the remaining 90 patients, 49 (54.4%) were in the sesame oil group and the rest of them in the placebo group (45.6%). The flow diagram of the study is demonstrated in Figure 1.

**Fig. 2:**
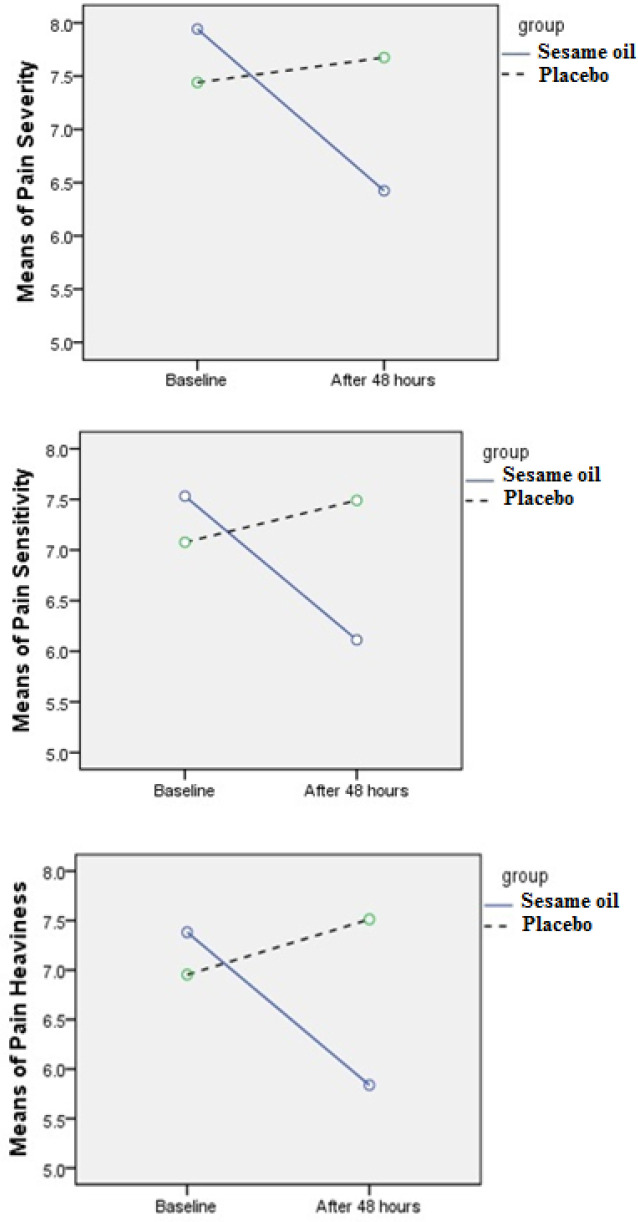
The changes of the pain severity, heaviness and sensitivity over time in the two study groups

The mean age of the patients was 28.3 ± 6.8 (ranging from 25 to 35) years and there were 63 (70%) men and 27 (30%) women among the patients. Both study groups were comparable regarding the baseline characteristics and the demographics (Table 1). In order to explore the effect of the interventions on pain severity, pain sensitivity and heaviness of painful site, we compared the baseline scores and those after 48 hours in each group separately. These results showed that the patients receiving sesame oil extracted from tahini felt less pain severity, pain sensitivity and heaviness of painful site than the other group (*p*<0.001). In addition, comparison of the mean differences of the baseline scores and those after 48 hours between the placebo and intervention groups showed a significant difference (Table 2). Figure 2 shows the changes of the pain severity, heaviness and sensitivity over time in the two study groups.

## Discussion

According to the results, sesame oil extracted from Tahini led to a significant decrease in pain severity, pain sensitivity and heaviness of painful site on the second day in the intervention group compared to the placebo one. The results also showed that in the placebo group the scores of pain severity and pain sensitivity changed significantly in the negative directions. Along with the pain relief property of sesame oil, we observed that this substance prevented skin discoloration due to bruising. In the literature review, we could only find two similar studies that evaluated the effect of sesame oil on trauma. Based on Nasiri *et al*.’s study, massage with topical sesame oil led to a significant reduction in pain severity of patients with limbs trauma. This study finding showed that on the 9th day of intervention, pain severity decreased 81% in the sesame oil group, while it decreased only 18% in the placebo group compared to the baseline (first day) [30]. The other study indicated that topical use of pure sesame oil reduced the pain severity of patients with upper or lower extremities trauma. In this research, the frequency of the received NSAIDs in the intervention and control groups was also recorded, showing a significant diﬀerence four days after the intervention [31]. Although these studies’ follow ups were more than 2 days, their final results were consistent with our findings. Most previous studies showed that the usage of sesame oil in combination with different extracts or sesame supplementation was effective in pain relief of patients with other problems like burn or knee osteoarthritis [32-35]. In Ang *et al*. and Hirsch et al.’s studies, the effect of an ointment containing sesame oil in combination with other herbal extracts and beta-Sitosterol on patients with burn was evaluated. It was shown that massage with moist exposed burn ointment (MEBO) decreased pain in these patients [33, 34]. Zahmatkash and Vafaeenasab demonstrated that the herbal ointment containing sesame oil, cinnamon, ginger, and mastic (Saghez) were effective for the pain relief of patients with knee osteoarthritis [32]. In another clinical trial study, the antinociceptive effect of amitriptyline for the lesions of the limb requiring primary suturing was investigated. From the first to the fifteenth minutes’ post-intervention, the mean score of pain in the amitriptyline group was significantly lower than the other group [36].

On the other hand, there were some animal studies that pointed to the analgesic properties of sesame oil (as topical or oral) in reducing pain due to different causes [37-42]. For example, in diabetic rats, usage of sesame oil can decrease the pain dysesthesia caused by diabetic peripheral circulatory disorders [39]. Also, oral consumption of sesame oil was effective on pain relief due to gout induced acute [38] and osteoarthritis-associated joint [40, 41].

Although the high content of unsaturated fatty acids (palmitic, stearic, oleic and linoleic acids), lignans (sesamin, asarinin, sesamolin and sesamol) and gamma-tocopherol responsible for pain relief are sesame oil benefits [43], the exact mechanism of action by which sesame oil alleviates pain is still unknown. Thus, exploring the mechanisms of action and correlating the pharmacological activity with chemical composition of sesame oil seem to be necessary. In this study, the limitation noted was the patients’ response to pain which is affected by genetic differences that were out of the researchers’ control. Also, we tried to consider most of the confounding factors by the inclusion and exclusion criteria. The other limitation was the small sample size and the single center experience. However, the study had an 80% power to detect the difference regarding the main study outcomes. Further larger and multi-center studies are required to elucidate the role of the sesame oil on pain severity in trauma patients. 

In conclusion, our findings suggest that the topical use of sesame oil extracted from Tahini has a pain reliever effect on the skin after bruising and it helps prevent skin discoloration. Further larger studies are required to confirm the results of the current study. 

**Table 1:** Baseline characteristics of the patients with limb trauma in the study groups

**Variables**		**Sesame oil (n=49)**	**Placebo (n=41)**	** P-value**
Age	15-25	9 (18.4%)	16 (39%)	0.093
	25-35	25 (51%)	15 (36.6%)	
	35-40	15 (30.6%)	10 (24.4%)	
Gender	Men	32 (65.3%)	31 (75.6%)	0.285
	Women	17 (34.7%)	10 (24.4%)	
Site of injury	Upper extremity	23 (47%)	14 (34.2%)	0.216
	Lower extremity	16 (32.6%)	21 (51.2%)	
	Both	10 (20.4%)	6 (14.6%)	

**Table 2:** Comparison of pain severity before and after the interventions in each group and also between groups

**Score**	**Group**	**Baseline**	**After 48 hours**	**diff**	**p-value **
**Pain ** **severity**	Sesame oil	7.94 ± 1.00	6.42 ± 1.00	-1.53 ± 0.57	<0.001
	Placebo	7.44 ± 1.32	7.68 ± 1.2	0.22 ± 0.52	0.001
**p-value (Independent t test)**				<0.001	
**Pain ** **Sensitivity**	Sesame oil	7.53 ± 1.24	6.11 ± 1.09	-1.45 ± 0.64	<0.001
	Placebo	7.07 ± 1.3	7.48 ± 1.24	0.39 ± 0.61	<0.001
**P-value (Independent t test)**				<0.001	
**Pain Heaviness**	Sesame oil	7.38 ± 1.17	5.83 ± 0.94	-1.56 ± 0.68	<0.001
	Placebo	6.91 ± 1.5	7.52 ± 1.24	0.5 ± 0.76	<0.001
**P-value (Independent t test)**				<0.001
